# Characterization of Corosolic Acid as a KPC-2 Inhibitor That Increases the Susceptibility of KPC-2-Positive Bacteria to Carbapenems

**DOI:** 10.3389/fphar.2020.01047

**Published:** 2020-07-09

**Authors:** Yonglin Zhou, Xiaohong Lv, Meishan Chen, Yan Guo, Rui Ding, Bin Liu, Xuming Deng, Jianfeng Wang

**Affiliations:** ^1^ Department of Respiratory Medicine, The First Hospital of Jilin University, Changchun, China; ^2^ Key Laboratory of Zoonosis, Ministry of Education, College of Veterinary Medicine, Institute of Zoonosis, Jilin University, Changchun, China; ^3^ Jilin Institute for Food Control, Changchun, China

**Keywords:** KPC-2, corosolic acid, carbapenems, anti-infection, *Enterobacteriaceae*

## Abstract

The emergence of KPC-producing Gram-negative bacteria in clinical practice highlights the need to search for novel antimicrobials and new anti-infection strategies. In this study, we constructed a laboratory KPC-2-positive strain, *E. coli* BL21(DE3) (pET28a-KPC-2) and identified the activity of KPC-2 in this strain. Using enzyme inhibition assays, checkerboard MIC assays, growth curves, time-killing assays and combined disk test, we found that the natural compound corosolic acid (CA) significantly inhibited the activity of the class A β-lactamase KPC-2, which is common among clinical isolates. CA treatment increased the antibacterial or bactericidal activity of imipenem and meropenem against *E. coli* BL21(DE3) (pET28a-KPC-2) *in vitro* (FIC index = 0.17 ± 0.03 for both carbapenems). In addition, the mouse intraperitoneal infection model confirmed that the combination therapy significantly reduced the bacterial load in the livers and spleens following subcutaneous administration. Our results showed that CA can be used to extend the life of carbapenems, providing a viable strategy for severe infections caused by KPC-2-positive bacteria.

## Introduction

The emergence of multidrug-resistant Gram-negative bacteria is one of the major challenges currently facing clinical medicine and livestock breeding. Multidrug-resistant Gram-negative bacteria, such as *Klebsiella* *pneumoniae* carbapenemase (KPC)-positive *Klebsiella pneumoniae* (*K. pneumoniae*), New Delhi metallo-β-lactamase (NDM)-positive and mobile colistin resistance (MCR)-positive *Escherichia coli* (*E. coli*), and multidrug-resistant *Acinetobacter baumannii* (*A. baumannii*), appear to have already spread worldwide (Patel and Bonomo, 2013; Wang et al., 2019). Of note, the emergence and spread of these multidrug-resistant Gram-negative bacteria is fuelled by the improper use of different antibiotics, the horizontal transmission of resistance determinants, suboptimal clinical medication regimen and the frequency of international exchange and cross-border travel, among other factors (Payne, 2016). To address this important issue, novel antibacterial agents, new treatment options or combination therapies are urgently needed to address serious Gram-negative pathogen infections (Liu et al., 2019).

The production of β-lactamases is one of the most common causes of resistance to β-lactam antibiotics in Gram-negative microorganisms. Serine-associated β-lactamases have been grouped into class A, class C and class D, and metallo β-lactamases have been grouped into class B (Bonomo, 2017; Bush, 2018). Among them, KPC-2 β-lactamase is a class A serine β-lactamase and is the most common cause of extreme resistance to β-lactam antibiotics in carbapenem-resistant *Enterobacteriaceae* (CRE) (Munoz-Price et al., 2013). Crucially, KPC-2 can hydrolyse all FDA-approved β-lactam antibiotics and β-lactamase inhibitors, such as sulbactam, tazobactam and clavulanic acid (Munoz-Price et al., 2013; Pemberton et al., 2017). Among the KPC variants, KPC-2 is the most widespread in *Enterobacteriaceae* in the United States and has shown a trend of global spread (Sanchez et al., 2013).

Corosolic acid (CA) is a pentacyclic triterpenoid that has been discovered in the extracts of leaves of *Eriobotrya japonica*, roots of *Actinidia chinensis* and fruits of *Crataegus pinnatifida* var. psilosa (Ulbricht et al., 2007; Miura et al., 2012). CA has been used for many years against diabetes in folk medicine in various animal models and humans without adverse effects (Miura et al., 2012; Ma et al., 2018). Furthermore, CA has also shown potential antioxidant, anti-inflammatory, antiviral, antibacterial, antifungal, antihyperlipidemic and antineoplastic activities and anti-proliferative activities in treatment (Shi et al., 2008; Stohs et al., 2012; Woo et al., 2018). For example, CA has been reported to increase glucose uptake in L6 myotubes and facilitate glucose transporter translocation in CHO/hIR cells by upregulating insulin receptor phosphorylation (Shi et al., 2008). In addition, CA increases the production of intracellular ROS, leading to the induction of apoptosis in lung adenocarcinoma cells, which is the key to the antitumor effect of CA. As a potential agent for the prevention and treatment of type 2 diabetes and obesity, CA has entered Phase III clinical pharmacodynamic evaluation of the Food and Drug Administration (FDA) in the USA (Ma et al., 2018).

Although CA has vast biological activity, it has not been reported as an inhibitor of β-lactamase (Sharma et al., 2018). Previous reports have indicated that enzyme inhibitors can restore the antibacterial activity of antibiotics *in vitro*/*in vivo*, so screening for effective inhibitors is a strategy to reverse antibiotic resistance (Zhou et al., 2018a; Zhou et al., 2018b). Towards this aim, we screened effective inhibitors of KPC-2 from natural compounds by enzyme inhibition assays. Herein, we discovered that CA, as an effective inhibitor of KPC-2, significantly restored the antibacterial activity of imipenem (IMP) and meropenem (MEP) against KPC-2-positive bacteria. In this study, we confirmed the synergistic effect of CA in combination with carbapenem antibiotics against KPC-2-positive bacterial infection *in vivo*/*in vitro* and laid the foundation for developing CA as a clinically available inhibitor.

## Materials and Methods

### Bacterial Strains and Chemicals

*E. coli* BL21(DE3) (pET28a-KPC-2) (preserved complete signal peptide) and *E. coli* BL21(DE3) (pET28a)(KPC-2) carried *kpc-2* (NCBI reference sequence: NG_049253.1) gene synthesized according to the sequences reported in NCBI (Smith Moland et al., 2003). In addition, *E. coli* BL21(DE3) (pET28a) was used as a negative control strain.

CA was purchased from Yuanye Bio-Technology Co., Ltd., Shanghai, China. Kanamycin, IMP and MEP were purchased from the National Institute for the Control of Pharmaceutical and Biological Products (Beijing, China). Nitrocefin (CAS: 41906-86-9) was purchased from TOKU-E Company, Bellingham, WA, USA. Dimethyl sulphoxide (DMSO), bacterial medium and all chemicals were obtained from Sigma Chemical Co. (St. Louis, MO, USA).

### Expression and Purification of Recombinant KPC-2

The *bla*_KPC-2_ gene was synthesized and cloned into pET28a to generate expression vectors. For expression of recombinant KPC-2, the forward primer was 5’-*ctgggatccatggcggaaccattcgcta*-3’ with a BamHI cut site, and the reverse primer was 5’-*ctggtcgacttactgcccgttgacg*-3’ with a XhoI cut site. For MIC assays, the forward primer was 5’-*ccgggatccatgtcactgtatcgccgt*-3’ with a BamHI cut site (preserved complete signal peptide), and the reverse primer was same with expression of KPC-2. First, the constructed expression vector was transferred into *E. coli* DH5α; the plasmid was extracted, and the correctness of *kpc-2* was verified. Then, the plasmid was transferred into *E. coli* BL21(DE3) to express the protein KPC-2.

The strain of *E. coli* BL21(DE3) (pET28a)(KPC-2) was cultured overnight in 500 ml of LB medium supplemented with 50 μg/ml kanamycin and grown to mid-logarithmic phase to OD_600 nm_ = 0.7 ± 0.1. Then, 1 mM isopropyl-β-D- thiogalactopyranoside (IPTG) was added to the bacterial culture, and the culture was induced at 15°C overnight. The bacterial cells were centrifugally harvested and resuspended in lysis buffer for sonication. A Ni column was used to remove protein impurities and unbound proteins and obtain pure KPC-2 protein. The post-dialysis protein was stored at −80°C.

### Detection of KPC-2 Activity Using the Carba NP Test

Detection of carbapenemase-positive bacteria using the Carba NP test was performed previously (Nordmann et al., 2012). The Carba NP test was also used in this study to detect KPC-2-positive bacteria. In brief, test solution A and solution B were prepared. A diluted phenol red solution was prepared by adding 2 ml of a phenol red solution (0.5%) to 16.6 ml of distilled water. Second, 360 ml of 10 mM ZnSO4 was added to the diluted phenol red solution, and then 0.1 M NaOH was used to adjust the pH value to 7.8 ± 0.1. The final solution was used as test solution A, and test solution A supplemented with 12.0 mg/ml imipenem and mixed thoroughly was used as test solution B.

The *E. coli* BL21(DE3) (pET28a-KPC-2) strain was incubated in LB medium (containing 50 μg/ml kanamycin and 70 μg/ml ZnSO4) with agitation at 37°C for 5 h. The bacteria were harvested by centrifugation, resuspended in 20 mM Tris–HCl lysis buffer and incubated for 30 min. Then, 100 μl of lytic bacterial solution was added to a 96-well plate with 100 μl of test solutions A and B in different wells and incubated at 37°C for 1–2 h. The color and absorbance changes in each well were detected.

### Enzyme Inhibition Assays

The β-lactamase activity was detected using nitrocefin as described previously (Zhou et al., 2020). In this study, it was first determined that CA inhibited the activity of KPC-2 during bacterial growth and in unprocessed culture supernatants. Briefly, mid-logarithmic-phase bacterial cells of *E. coli* BL21(DE3) (pET28a-KPC-2) were diluted in LB medium supplemented with different concentrations of CA for 6 h at 37°C, and the culture supernatants were harvested by centrifugation (10,000 rpm) at 4°C for 10 min. Then, 100 μl of the supernatant was mixed with 75 μl of phosphate-buffered saline (PBS) and 25 μl of diluted nitrocefin, and the mixture was incubated at 37°C for 30 min. The change in absorbance at 492 nm and color changes of the solution were determined at room temperature. In addition, the β-lactamase activities were determined by CA treatment of the supernatants from the untreated bacterial culture and purification of KPC-2 as described above. The software of Graphpad Prism was powerful enough to help calculate the IC_50_ (half maximal inhibitory concentration) value. The minimum concentration of CA required for inhibiting the activity of purification of KPC-2 to be half is the IC_50_. First, a series of concentration gradients of CA (0–64 μg/ml) were set to determine the percentage of enzyme activity inhibition of KPC-2. All tests were determined in triplicate. Then, the IC50 value can be calculated using Graphpad prism.

### Checkerboard MIC Assays and MIC Assays

The MICs for the strain of *E. coli* BL21(DE3) (pET28a-KPC-2) were determined with the two-fold checkerboard microdilution method essentially as described in the Clinical and Laboratory Standards Institute (CLSI) protocol (Wiegand et al., 2008; Xu et al., 2012). Briefly, two-fold dilutions of CA (final concentrations ranging from 0 to 128 μg/ml in 100 μl of LB medium) were added to each column, and two-fold dilutions of carbapenem antibiotics (final concentrations of IMP ranging from 0 to 64 μg/ml and MEP ranging from 0 to 16 μg/ml in 100 μl of LB medium) were added in each row in a 96-well plate. Then, 100 μl of 1 × 10^6^ CFU/ml bacterial cells were inoculated and grown for 24 h at 37°C. The MIC values of CA or carbapenem antibiotics were determined by the turbidity of each well based on visual inspection. For the MIC test, two rows of treatments, the tested antibiotics with or without CA (32 μg/ml), were performed. The fractional inhibitory concentration (FIC) index values were calculated by the formula: FIC index = (MIC _CA used alone_/MIC_CA used in combination_) + (MIC_antibiotics used alone_/MIC_antibiotics used in combination_). Synergy, FIC index ≤0.5; addition effect, FIC index >1; and invalid, 0.5 < FIC index ≤ 1.

### Growth Curves

Growth curves were used to determine the influence of CA on the growth of *E. coli* BL21(DE3) (pET28a-KPC-2) (Zhou et al., 2018b). Initially, overnight cultured bacterial cells were diluted into fresh LB medium and grown at 37°C to a starting OD600 of 0.3. The bacterial cultures of *E. coli* BL21(DE3) (pET28a-KPC-2) were transferred into six Erlenmeyer flasks with different concentrations of CA (from 0 to 64 μg/ml). Then, the bacteria in each Erlenmeyer flask were cultured at 37°C with shaking under aerobic conditions, and each culture of bacteria was monitored by measuring the OD600 value every 30 min.

### Time-Killing Assays

The time-killing assays were performed as described in our previous study (Zhou et al., 2019). Bacterial strains of *E. coli* BL21(DE3) (pET28a-KPC-2) were cultured to logarithmic phase and diluted to 5 × 10^5^ CFU/ml in LB medium in each well of a 96-well plate. Then, CA (32 μg/ml), IMP (2 μg/ml) and IMP (2 μg/ml) in combination with CA (32 μg/ml) were added to different wells and statically cultured consecutively at 37°C. A ten-fold serial dilution method was used to detect the number of bacteria in each group at the appropriate timepoint (from 0 to 24 h). The different samples of each group were coated onto kanamycin LB agar plates. The number of colonies of each sample was recorded after incubation overnight at 37°C.

### Combined Disk Test

The combined disk test was also used to determine the synergy of CA and carbapenem antibiotics (Boonyanugomol et al., 2017; Zhou et al., 2019). According to our previous report and the MIC value of CA against *E. coli* BL21(DE3) (pET28a-KPC-2), 10 μl of DMSO containing different concentrations of CA (final concentrations were 0, 8 and 32 μg/mL) was mixed into warm LB medium with agar (0.7%) to produce LB agar plates. Each bacterium diluted to OD600 = 0.1 was inoculated on LB agar plates. Then, a disk containing 10 μg of MEP (Oxoid Ltd., Basingstoke, UK) was placed on the centre of LB agar plates. The inhibition zone diameters of the MEP disks on the LB agar plates were measured and photographed after incubation for 18–24 h at 37°C. *E. coli* BL21(DE3) (pET28a) was used as a positive control.

### Mouse Intraperitoneal Infection Model

Male C57BL/6J mice were 6–8 weeks old and were purchased from Changsheng Biotechnology Co., Ltd., Shenyang, China. The animal experiments were approved by the guidelines of the Animal Care and Use Committee of Jilin University, and the guidelines were strictly followed.

The mice were anesthetized and intraperitoneally infected with 3 × 10^8^ CFU of *E. coli* BL21(DE3) (pET28a-KPC-2) to cause an intraperitoneal infection. All the infected mice were randomly divided into four groups: subcutaneously administered IMP (20 mg/kg), CA (25 mg/kg), IMP (20 mg/kg) combined with CA (25 mg/kg) and control solvent. All mice were administered every 12 h up to 2 days and then were sacrificed after anesthesia. The liver and spleen from different groups of mice were weighed, homogenized and plated on kanamycin-resistant LB agar plates for calculation of bacterial load.

### Statistical Analysis

The experimental data were analysed by the software Statistical Program for Social Sciences (SPSS), and the data were finally expressed as the mean ± standard deviation. The IC_50_ of CA for inhibition of KPC-2 was calculated by software GraphPad Prism 5.01. Significant differences were determined using an independent Student’s T test. P values ≤0.05 were considered significant, and P values ≤0.01 were considered extremely significant.

## Results

### CA Significantly Inhibited the Enzymatic Activity of KPC-2

**Figure 1:**
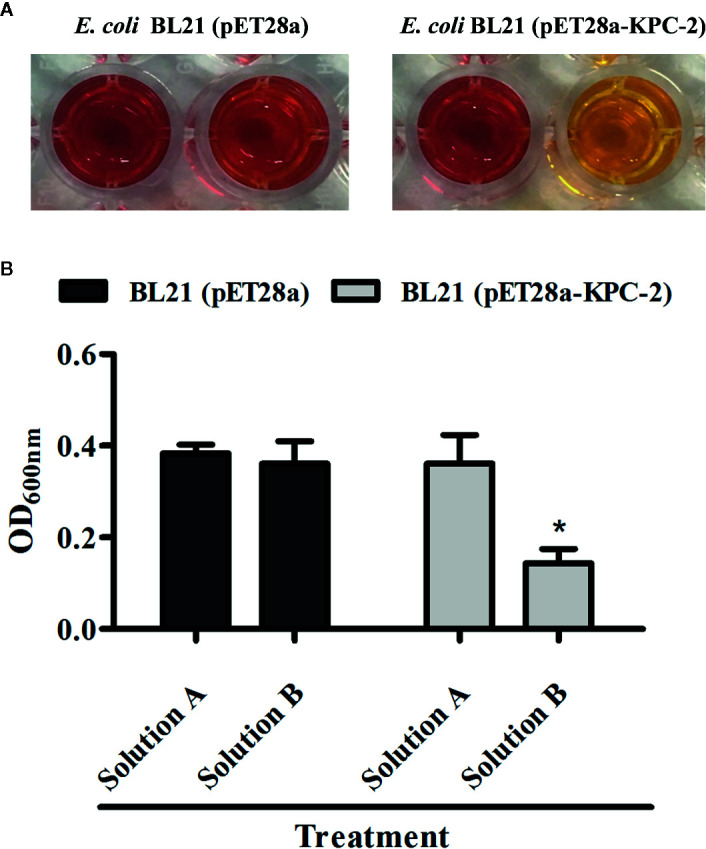
Identification of the activity of KPC-2 in constructed bacterial strains. The strains *E. coli* BL21 (pET28a-KPC-2) and *E. coli* BL21 (pET28a) were used for the Carba NP test. The results were evaluated by the color changes **(A)** and absorbance **(B)** at 600 nm. Solution A supplemented with imipenem (12.0 mg/ml) was used as test solution B. *indicates P < 0.05.

**Figure 2:**
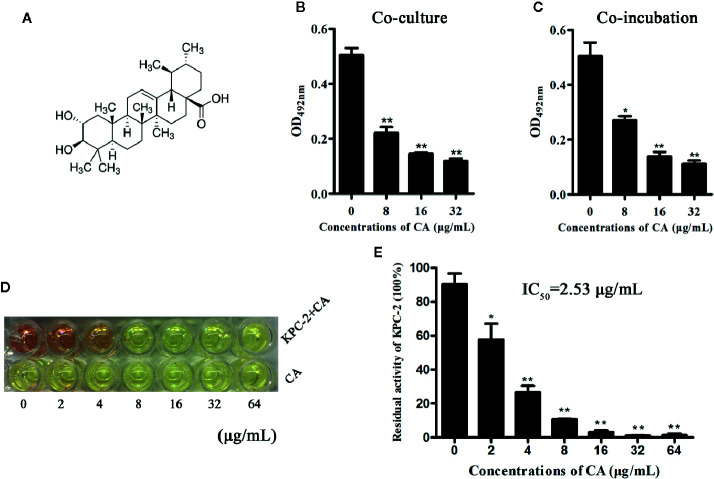
CA inhibited the activity of KPC-2. The structure of CA **(A)**. A significant inhibitory effect of CA on the activities of KPC-2 was detected by enzyme inhibition assays following co-culture **(B)** or co-incubation **(C)** with the indicated concentrations of CA (0–32 µg/ml). In addition, the activities of purified KPC-2 protein were also inhibited by CA in a concentration-dependent manner **(E)**. The color of the test solution gradually turned yellow as the concentration of CA increased, and CA itself did not cause color changes **(D)**. **indicates P < 0.01; *indicates P < 0.05.

The Carba NP test was used to determine whether the KPC-2-positive *E. coli* strain BL21(DE3) (pET28a-KPC-2) had the activity of carbapenemase-positive bacteria before screening KPC-2 inhibitors. As shown in **Figure 1**, Carba NP test solution B with *E. coli* BL21(DE3) (pET28a-KPC-2) turned yellow, and solution A with *E. coli* BL21(DE3) (pET28a-KPC-2) turned crimson, while solutions A and B with *E. coli* BL21(DE3) (pET28a) were crimson (**Figure 1A**). Meanwhile, the absorbance at OD600 nm of solution B with *E. coli* BL21(DE3) (pET28a-KPC-2) was also significantly reduced compared with that of solution A with *E. coli* BL21(DE3) (pET28a-KPC-2) (**Figure 1B**). Then, enzyme inhibition assays were used to detect the activities of KPC-2 in bacterial culture supernatants when co-cultured or co-incubated with different concentrations of CA. CA (**Figure 2A**) exerted a significant inhibitory effect against the activity of KPC-2 when co-cultured with *E. coli* strain BL21(DE3) (pET28a-KPC-2) or co-incubated in culture supernatants (**Figures 2B, C**). In addition, it was also confirmed that CA had a significant inhibitory effect on purified KPC-2 (**Figures 2C, D**). The IC_50_ of CA for inhibition of KPC-2 was 2.35 µg/ml.

### CA Showed a Synergistic Effect Against *E. coli* BL21(DE3) (pET28a-KPC-2) When Combined With Carbapenems

**Figure 3:**
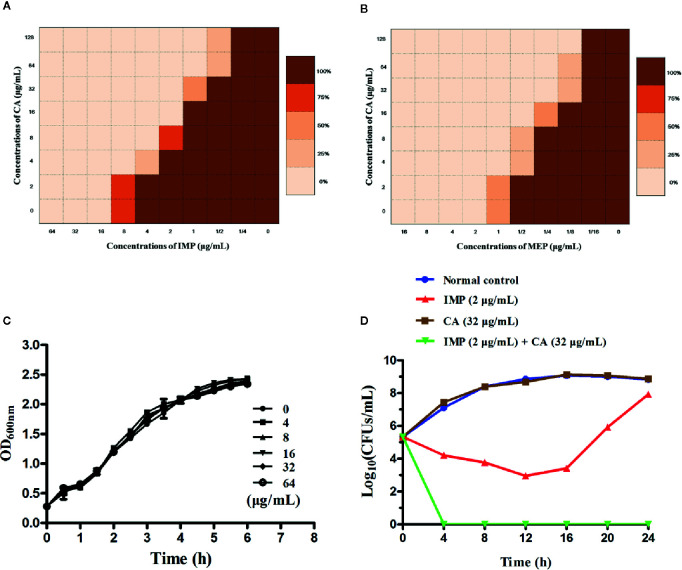
Checkerboard MIC analysis showed a synergistic effect of CA combined with IMP **(A)** or MEP **(B)** against the carbapenemase-positive laboratory strain *E. coli* BL21 (pET28a-KPC-2). Growth curves for *E. coli* BL21(DE3) (pET28a-KPC-2) **(C)**. Time-killing curves for the CA, IMP, CA and IMP in combination and control treatments (medium only) against *E. coli* BL21(DE3) (pET28a-KPC-2) **(D)**.

We applied checkerboard MIC assays and MIC assays to identify potential synergies between CA and carbapenems (including IMP and MEP). The results of the checkerboard MIC tests with *E. coli* BL21(DE3) (pET28a-KPC-2) showed that CA, at concentrations of CA ≥32 µg/ml, led to the highest MIC fold change of ≥16 for both IMP and MEP, and the FIC index values of this combination were all less than 0.5 (**Figures 3A, B**, **Table 1**). No synergy was observed in the KPC-2-negative strain *E. coli* BL21(DE3) (pET28a) when CA was used in combination with carbapenems (**Table 1**). Moreover, the growth curve results showed that CA (0–64 µg/ml) did not affect the growth of *E. coli* BL21(DE3) (pET28a-KPC-2) (**Figure 3C**).

**Table 1:** MIC values of the carbapenems and CA combination therapy for each of the tested bacterial isolates.

Species	Antibiotics	MIC of Antibiotics (μg/mL)		FIC Index
		Alone	Combination	
***E. coli* BL21(DE3)** **(pET28a-KPC-2)**	Meropenem	14.00 ± 4.00	1.5 ± 0.58	**0.17 ± 0.03**
	Imipenem	1.50 ± 0.58	0.16 ± 0.06	**0.17 ± 0.03**
***E. coli* BL21(DE3)** **(pET28a)**	Meropenem	0.008 ± 0.00	0.008 ± 0.00	1.06 ± 0.00
	Imipenem	0.094 ± 0.04	0.078 ± 0.031	0.94 ± 0.25

The concentration of CA was 32 μg/ml in E. coli BL21(DE3) (pET28a-KPC-2) and E. coli BL21(DE3) (pET28a). The FIC values of KPC-2-positive bacterial isolates were indicated in bold. The data were presented as the mean ± standard deviation.

**Figure 4:**
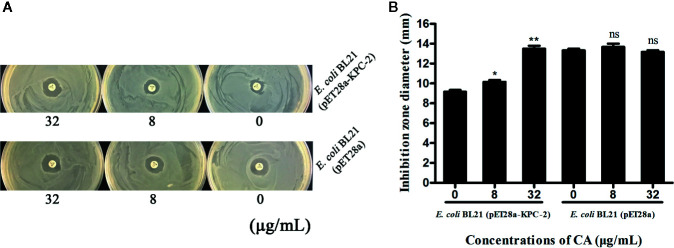
Zones of inhibition surrounding MEP disks on LB agar plates with different concentrations of CA (0, 8 and 32 µg/ml) **(A)**. The results of the combined disk test showed increases in the zones of inhibition surrounding MEP disks in a dose-dependent manner only for *E. coli* BL21(DE3) (pET28a-KPC-2) with different concentrations of CA **(B)**. **indicates P <0.01; *indicates P < 0.05; ns indicates no significant difference.

Time-killing assays and a combined disk test were used to further confirm the synergistic effect between CA and carbapenems. The bacteria could not be killed when CA (32 µg/ml) or IMP (2 µg/ml) were used alone. In contrast, the combination of CA (32 µg/ml) and IMP (2 µg/ml) resulted in the elimination of *E. coli* BL21(DE3) (pET28a-KPC-2) at 3 h post-administration (**Figure 3D**). The results of the combined disk test showed that the zones of inhibition around the MEP disks increased in a dose-dependent manner for *E. coli* BL21(DE3) (pET28a-KPC-2) with different concentrations of CA, while the zones of inhibition of *E. coli* BL21(DE3) (pET28a) with CA did not significantly increase (**Figures 4A, B**).

### Combination Therapy of CA and IMP Had a Synergistic Effect In Vivo

**Figure 5:**
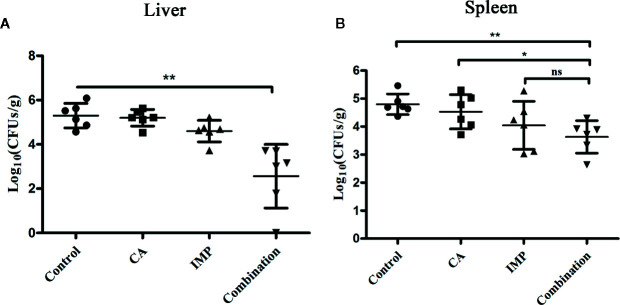
Confirmation of the synergistic effect of CA in combination with IMP *in vivo*. The mice were intraperitoneally administered *E. coli* BL21(DE3) (pET28a-KPC-2), and the bacterial burden of the livers **(A)** and spleens **(B)** was calculated. **indicates P < 0.01; *indicates P < 0.05; ns indicates no significant difference.

Due to the limited number of experimental mice, we only assessed the effect of therapy on the bacterial load in the livers and spleens. CA in combination with IMP resulted in a significant reduction in the bacterial load in the livers compared with the monotherapy treatments and the control (P < 0.01) (**Figure 5A**). The combination therapy also showed a significant decrease in bacterial load in the spleens compared with the control group and CA treatment, although it only showed a decrease in the number of CFU compared with IMP treatment (**Figure 5B**).

## Discussion

Gram-negative bacteria carrying KPC-2 mainly include *K. pneumoniae*, *E. coli*, *A. baumannii* and *P. aeruginosa*, with the highest prevalence of KPC-2 among *K. pneumoniae*. *K. pneumoniae* is the most common KPC-2-carrying bacterium in several Asian countries and causes many invasive diseases, such as pneumonia and meningitis (Moon et al., 2010; Shon et al., 2013). A study by Xu showed a high prevalence and mortality of KPC-2-positive *K. pneumoniae* causing meningitis in eastern China, posing a serious threat to public health (Xu et al., 2019). It has been reported that hundreds of compounds can significantly inhibit the activities of NDM-1 *in vitro*/*in vivo*, while only a few KPC-2 inhibitors have been discovered, and most inhibitors are pre-reported β-lactamase inhibitors; for example, avibactam obtained through chemical modification (Durand-Réville et al., 2007; King et al., 2014; Liu et al., 2018). Therefore, our research is urgently needed to treat KPC-2-positive bacterial infections in the clinic.

Pentacyclic triterpenoids usually have similar or identical biological activities, and polycyclic terpene acids such as oleanolic acid may also contribute to antihyperglycaemic, antiviral and other pharmacodynamic effects similar to the effects of CA (Eldridge et al., 2002; Garo et al., 2007). Our previous research revealed that oleanolic acid can significantly inhibit most carbapenemases, including NDM-1 and KPC-2 (data not shown). Therefore, we speculated that CA can significantly inhibit the activities of other carbapenemases and had significant synergy with β-lactam antibiotics against carbapenem-resistant *Enterobacteriaceae* (CRE).

The aqueous extract of banaba or CA has been used in folk medicine to decrease blood glucose levels for a long time (Choi et al., 2010; Miura et al., 2012). CA has been shown to have a significant effect in the treatment of various animal models and human diseases, especially diabetes mellitus, without adverse effects (Sivakumar et al., 2009). It was a valuable result that no significant symptoms of poisoning were found in mice treated with CA in this study. Further studies, including the design of the dosing regimen, mode of administration and dosage of CA, are needed to optimize the effects of combination therapy.

In summary, the synergistic effect of CA combined with carbapenem antibiotics was demonstrated in a strain carrying KPC-2. Therefore, our research provided a workable strategy for the treatment of KPC-2-positive bacterial infections.

## Data Availability Statement

The raw data supporting the conclusions of this article will be made available by the authors, without undue reservation, to any qualified researcher.

## Ethics Statement

The animal study was reviewed and approved by the Animal Care and Use Committee of Jilin University.

## Author Contributions

Study design: JW, XD, YZ. Experimental studies: YZ, XL, MC, BL. Data analysis/interpretation: YG, RD. Statistical analysis: YZ, XL. Manuscript preparation: JW, XD, YZ.

## Funding

This work was supported by the National Key Research and Development Program of China (no. 2018YFD0500300), and the National Natural Science Foundation of China (grant 81861138046).

## Conflict of Interest

The authors declare that the research was conducted in the absence of any commercial or financial relationships that could be construed as a potential conflict of interest.
